# Genomic Insights into Global *bla*_CTX-M-55_-Positive Escherichia coli Epidemiology and Transmission Characteristics

**DOI:** 10.1128/spectrum.01089-23

**Published:** 2023-06-26

**Authors:** Jin-Tao Yang, Li-Juan Zhang, Yang Lu, Rong-Min Zhang, Hong-Xia Jiang

**Affiliations:** a Guangdong Laboratory for Lingnan Modern Agriculture, South China Agricultural University, Guangzhou, China; b Guangdong Key Laboratory for Veterinary Pharmaceutics Development and Safety evaluation, College of Veterinary Medicine, South China Agricultural University, Guangzhou, China; c Zhaoqing Branch Center of Guangdong Laboratory for Lingnan Modern Agricultural Science and Technology, Zhaoqing, China; Instituto de Higiene

**Keywords:** *bla*_CTX-M-55_, *Escherichia coli*, whole-genome sequencing, plasmid

## Abstract

In recent years, *bla*_CTX-M-55_-positive Escherichia coli has been widely reported in multiple locations with an increasing trend in prevalence, yet few studies have comprehensively analyzed the transmission characteristics and epidemiological patterns of *bla*_CTX-M-55_-positive E. coli. Here, we constructed a *bla*_CTX-M-55_-positive E. coli global genomic data set as completely as possible and explored the epidemiology and potential impact of *bla*_CTX-M-55_-positive E. coli on a global scale by high-resolution bioinformatics methods. The results show that *bla*_CTX-M-55_-positive E. coli has spread widely worldwide, especially in Asia, with the rich sequence typing (ST) diversity and high proportion of auxiliary genome occupancy indicating a high degree of openness. The phylogenetic tree suggests that *bla*_CTX-M-55_-positive E. coli is frequently clonally transmitted between the three human-animal environments and often cotransmitted with *fosA*, mcr, *bla*_NDM_, and *tet*(X). The stable presence of InclI1 and InclI2 in different hosts from different sources suggests that this part of the plasmid drives the widespread transmission of *bla*_CTX-M-55_-positive E. coli. We inductively clustered all *bla*_CTX-M-55_ flanking environmental gene structures and obtained five types. Notably, “IS*Ecp1*-*bla*_CTX-M-55_-*orf477*-(Tn2)” and “IS*26*(IS*15DI*)-*hp*-*hp*-*bla*_CTX-M-55_-*orf477*-*hp*-*bla*_TEM_-IS*26*-*hp*-IS*26*-Tn2” are dominant in “humans” and in “animals and related foods,” respectively. Overall, our findings highlight the importance of whole-genome sequencing-based surveillance in exploring the transmission and evolution of *bla*_CTX-M-55_-positive E. coli in the context of “One Health,” and they serve as a reminder to strengthen the surveillance of *bla*_CTX-M-55_-positive E. coli in order to address the potential risk of future large outbreaks.

**IMPORTANCE** CTX-M-55 was first discovered in Thailand in 2004, and today, this enzyme is the most common CTX-M subtype in E. coli of animal origin in China. Thus, *bla*_CTX-M-55_-positive E. coli getting widely spread is a growing public health problem. Although prevalence surveys of *bla*_CTX-M-55_-positive E. coli in different hosts have been widely reported in recent years, they remain insufficient in “One Health” context and from a global comprehensive perspective. Here, we constructed a genomic database of 2144 *bla*_CTX-M-55_-positive E. coli and used bioinformatics methods to resolve the spread and evolution of *bla*_CTX-M-55_-positive E. coli. The results suggest a potential risk of rapid transmission of *bla*_CTX-M-55_-positive E. coli and that long-term continuous surveillance of *bla*_CTX-M-55_-positive E. coli should be emphasized.

## INTRODUCTION

The World Health Organization (WHO) has identified antimicrobial drug resistance as one of the major threats to human public health and food safety in the 21st century (1). Multidrug resistant (MDR) enteric bacteria have spread globally, with extended-spectrum beta-lactamase (ESBL)-producing *Enterobacteriaceae* being the most common type (2). Among them, CTX-M is considered the most predominant enzyme in the widespread dissemination of ESBLs (3). At present, CTX-M-15 is the most popular CTX-M worldwide and the most common CTX-M subtype in Chinese clinical medicine (4, 5). CTX-M-55 and CTX-M-15 are both members of the CTX-M-1 group. The amino acid similarity between CTX-M-55 and CTX-M-15 is 99.3%, and the genetic relationship is the closest. Only a single amino acid, Ala-77-Val, is substituted in CTX-M-55. Therefore, CTX-M-55 is considered a derivative of CTX-M-15. In addition, CTX-M-55 showed stronger hydrolytic activity than CTX-M-15 against ceftazidime (4, 6). However, CTX-M-55 has been widely reported in Escherichia coli isolated from food animals and pets in the Chinese mainland and Hong Kong since it was first discovered in ESBLs produced in Thailand in 2004 (6). In addition, after CTX-M-55 was first detected in Chinese clinical clinics in 2010, the reports of this enzyme in China increased rapidly, and it has surpassed CTX-M-15 and become the second most common CTX-M subtype in Chinese clinical isolates of E. coli after CTX-M-14. It is also the most common CTX-M subtype in E. coli of animal origin in China (7–9).

Studies on the localization of *bla*_CTX-M_ to chromosomes have appeared several times in recent years (10), but plasmids are still considered to be the main reason for the rapid spread of *bla*_CTX-M_. Currently, the *bla*_CTX-M-55_ gene is mostly reported to be localized to the IncFII, IncI1, IncI2 and IncHI2 plasmids (11). In addition, *bla*_CTX-M_ is also associated with many insertion sequences (ISs), such as IS*Ecp1* and IS*26* (12), which further facilitates their dissemination. In addition, *bla*_CTX-M_ usually appears to colocalize with other antimicrobial resistance genes (ARGs), such as *fosA*, *mcr*, and *bla*_NDM_ (13, 14), which poses a great challenge for the clinic.

Although prevalence surveys of *bla*_CTX-M-55_-positive E. coli in different hosts have been widely reported in recent years, the analysis of *bla*_CTX-M-55_-positive E. coli is limited by geographical and temporal preferences and remains limited from a comprehensive global perspective. Therefore, for the first time, we constructed a genomic database of 2144 *bla*_CTX-M-55_-positive E. coli, a collection of genomes, including different times (2002 to 2022), different regions (Asia, North America, Europe, South America, Oceania, and Africa) and different sample origins (humans, animals and related foods, environment, and plants and related foods), and comprehensively elaborated the transmission and evolution of *bla*_CTX-M-55_-positive E. coli using genomic analysis methods.

## RESULTS

### Global distribution and high diversity of *bla*_CTX-M-55_-positive Escherichia coli.

**FIG 1:**
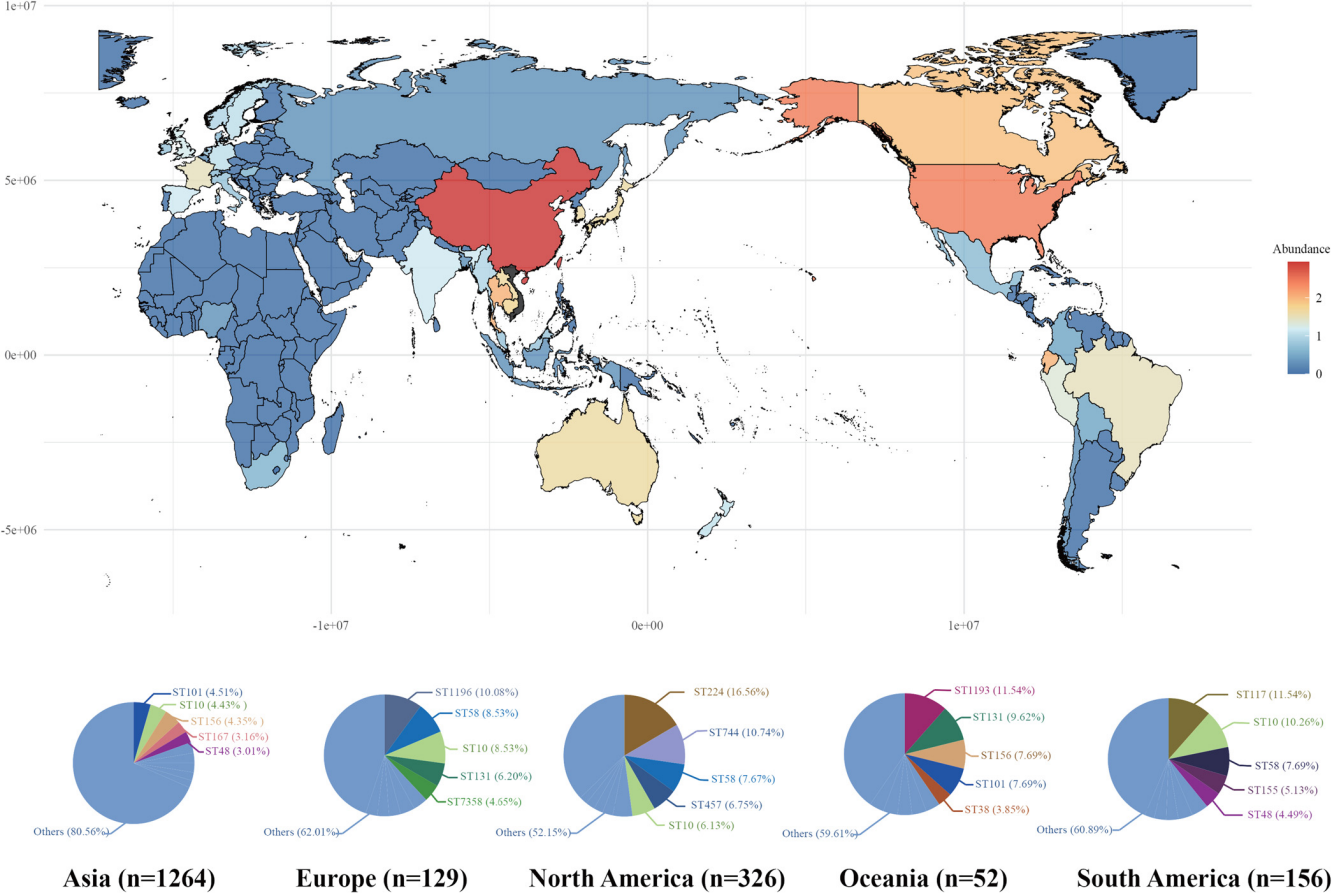
Global distribution of *bla*_CTX-M-55_-positive E. coli and distribution characteristics of the top 5 STs on different continents.

A total of 2144 *bla*_CTX-M-55_-positive E. coli genomes were collected in this study (66 from our laboratory and 2078 from the National Center for Biotechnology Information database as of September 2022). Overall, *bla*_CTX-M-55_-positive E. coli has spread to various degrees worldwide (except Antarctica) and is endemic almost worldwide, with the most severe epidemics in regions such as China (*n* = 878), the United States (*n* = 240), and Thailand (*n* = 101) (Fig. 1). Overall, multilocus sequence typing assigned them to 383 different sequence types (STs) (except for 66 isolates for which an allelic combination could not be assigned to a known ST), with an overall more dispersed distribution and high diversity. More than half of these STs (*n* = 218, 56.92%) were represented by single isolates, and most of them were present in Asia (Fig. S1). In addition, ST10 (*n* = 114, 5.3%) had the highest detection rate, and other major STs included ST224 (*n* = 86, 4.0%), ST156 (*n* = 80, 3.7%), ST744 (*n* = 71, 3.3%), and ST101 (*n* = 67, 3.1%). Similarly, the distribution of STs varied between regions, with the most prevalent ST in Asia being ST101, while the most prevalent STs in Europe, North America, Oceania, and South America were ST1196, ST224, ST1193, and ST117, respectively (Fig. 1 and Table S2). Of interest is that ST167 and ST1196 are more prevalent in the early years and decrease in frequency year by year; in contrast, ST10, ST156, and ST155 appeared more commonly in recent years (Fig. S2).

To better understand the openness/plasticity of strain 2144 of *bla*_CTX-M-55_-positive E. coli genomes, we performed a pangenomic analysis. The results demonstrated an open pangenome of 11,026 genes, but interestingly, its core genome progressed to 1.24% of all genes (*n* = 1365) and the pangenome was calculated by Heaps' law to get an alpha of 0.99 (<1.0). These results indicate that the pangenome possesses a high degree of openness. This result that could be related to its assignment to 383 different STs as described above. Again, the number of genes did not reach a steady state, suggesting that the number of genes still increased with sample size (Fig. S3).

### Phylogenetic analysis.

**FIG 2:**
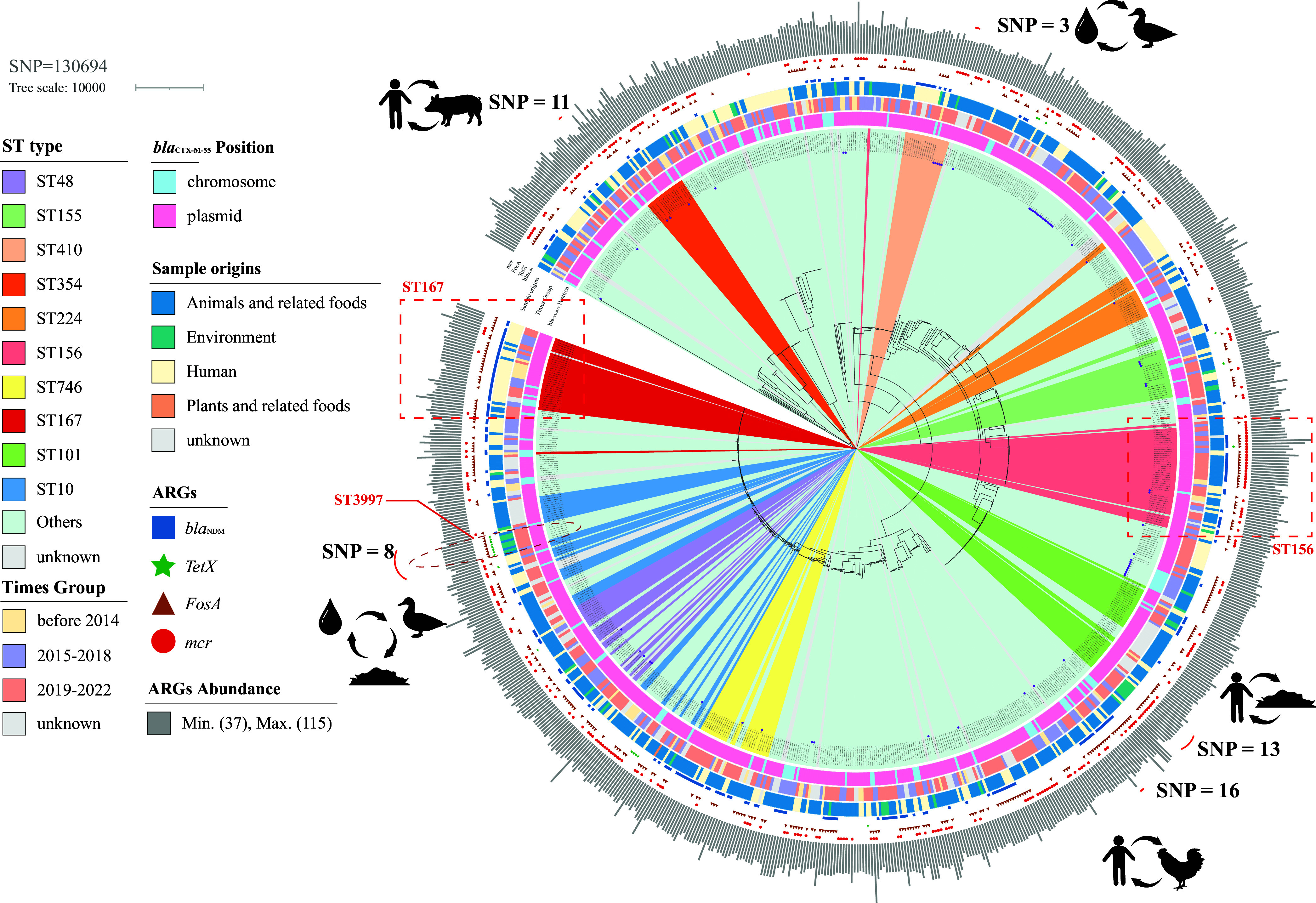
Phylogenetic tree of 878 *bla*_CTX-M-55_-positive E. coli isolates in China. The midpoint-rooted tree was constructed using *n* = 130,694 core genome SNPs.

Since *bla*_CTX-M-55_-positive E. coli is most prevalent in China, we subjected 878 regional Chinese isolates to a core genome phylogenetic analysis. In this study, we constructed a maximum likelihood phylogenetic tree based on 130,694 core genomic SNP loci to explore the spread and evolution of *bla*_CTX-M-55_-positive E. coli transmission in China. According to rfplasmid predictions, the vast majority of *bla*_CTX-M-55_ were predicted to be localized in plasmids (*n* = 761, 86.7%), and conversely, a small proportion was present on the chromosome (*n* = 117, 13.3%). In the present study, a close phylogenetic relationship existed between the laboratory isolates from Guangdong and the publicly collected isolates from China. Moreover, we usually define strains with ≤ 20 SNPs as clonal strains (15). Interestingly, clonal transmission was found in this study between several different media, such as human and pig (SNP = 11), human and chicken (SNP = 16), human and environment (SNP = 13), and water, duck and soil (SNP = 8), suggesting that *bla*_CTX-M-55_-positive E. coli has been widely spread among humans, animals and the environment (Table S3). Interestingly, in the human–chicken clonal transmission phenomenon described above, we observed that the human sample was from 2014 and the chicken sample was from 2016. We also show the coexistence of 4 important ARGs in *bla*_CTX-M-55_-positive E. coli, namely, *bla*_NDM_ (*n* = 235, 26.8%), *mcr* (*n* = 292, 33.3%), *tet*(X) (*n* = 21, 2.4%) and *fosA* (*n* = 395, 45.0%) (Fig. 2). Interestingly, we observed that certain specific STs preferentially carried specific ARGs. In ST167, *bla*_CTX-M-55_-positive E. coli usually coexists with both *bla*_NDM_ and *fosA* (*n* = 23, 69.7%) and tends to be carried by humans (*n* = 29,87.9%). In contrast, ST156 is frequently isolated from animals and related foods, and this ST is often found to carry *bla*_CTX-M-55_, *bla*_NDM_, *fosA* and *mcr* (*n* = 18,39.1%). In addition, all ST3997 samples in this study carried *fosA3* and *tet*(X4), which is of concern despite the clonal transmission of this part of the isolates (Fig. S4).

### ARG profiles.

**FIG 3:**
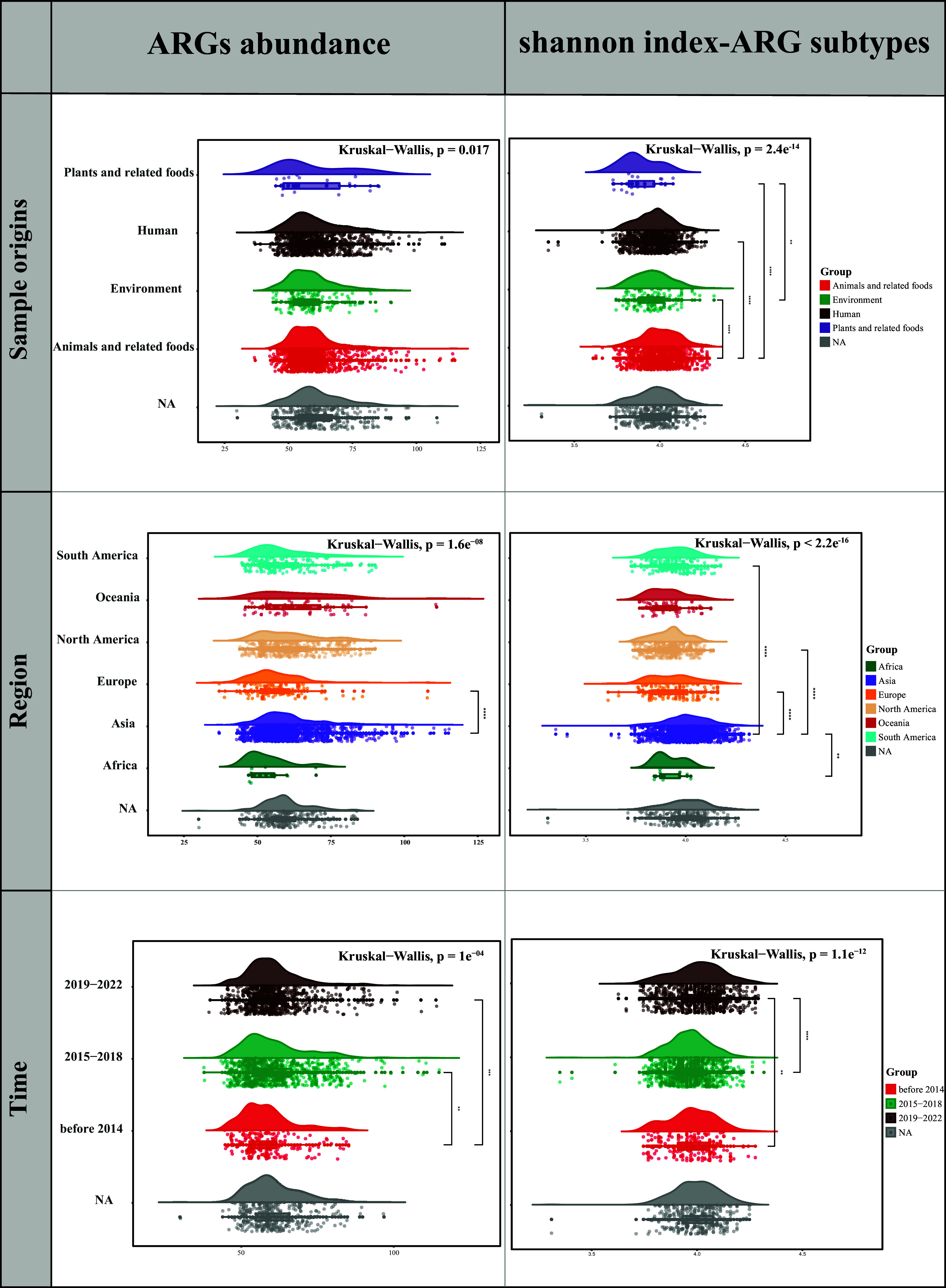
The distribution of ARG abundance and Shannon index-ARG subtypes for different sample origins, regions, and time groupings, where the asterisks on the right indicate the *P* value obtained using the Wilcoxon tests. (*, *P* < 0.05, **, *P* < 0.01, ***, *P* < 0.001, ****, *P* < 0.0001).

Overall, we identified a total of 258 ARG subtypes belonging to 16 ARG types in 2144 *bla*_CTX-M-55_-positive E. coli. Among all isolates, the most common ARG type was multidrug (43.4%) (Fig. S5 and Table S4). We sought to further understand the differences in the overall abundance and alpha diversity of ARGs in *bla*_CTX-M-55_-positive E. coli genomes at different grouping levels. Grouped according to different sample origins, the alpha diversity of ARG subtypes in animals and related foods were all higher than those in plants and related foods, humans, and the environment (*P* < 0.001), but they did not differ significantly in ARG abundance. Grouped by different regions, the alpha diversity of ARG subtypes was generally higher in Asia than on other continents (except Oceania) (*P* < 0.01), and it is also noteworthy that the ARG abundance was significantly higher in Asia than in Europe. Furthermore, according to different temporal groupings, ARG abundance in the pre-2014 group was lower than that in the 2015 to 2018 group and 2019 to 2022 group (*P* < 0.01), and alpha diversity was the highest in the 2019 to 2022 group (*P* < 0.01) (Fig. 3). In general, the abundance of ARGs and the alpha diversity of ARG subtypes showed an overall increasing trend over a time of almost 20 years. To further understand the beta diversity of ARG subtypes in different subgroups, PCoA was performed, the results of which showed significant grouping based on sample origin (ANOSIM, *P* = 0.001), region (ANOSIM, *P* = 0.001), and time (ANOSIM, *P* = 0.001) (Fig. S6). However, it was difficult to observe clear grouping aggregation boundaries in the plot, which may be due to the diversity of sample ranges.

### Correlations between ARG, MGE and virulence gene profiles.

To better understand the potential associations between ARGs, mobile genetic elements (MGEs) (ISs and plasmids) and virulence genes, we performed correlation analysis by the R package “ggstatsplot.” We observed a moderate correlation between ARG counts and IS counts (Spearman = 0.35, *P* < 0.001). Similarly, there was a weak positive correlation between plasmid replicon counts and ARGs (Spearman = 0.25, *P* < 0.001) but almost no correlation between ARG counts and VF counts (Spearman = −0.05, *P* = 0.02) (Fig. S7).

In addition, we defined *bla*_CTX-M-55_ as colocalized with ARGs and MGEs (ISs and plasmids) in the nearby region if they shared an overlapping cluster (<10 kb). Network visualization was performed by colocalization information, which can reflect to some extent the cotransfer and potential transfer patterns with *bla*_CTX-M-55_. The results show fewer connections in the colocated network until 2014, which become increasingly complex at later stages. We found that *bla*_CTX-M-55_ often coexists with *bla*_TEM-1_, *bla*_TEM-141_, *QnrS1* and *fosA3*. Similarly, we found closely linked colocalized MGEs for different geographical regions and sample sources, and this fraction of MGEs played an important role in the widespread distribution of *bla*_CTX-M-55_, such as IS*Ecp1*, IS*26*, IncI1, and IncI2 (Fig. S8).

### Sequence analysis of *bla*_CTX-M-55_-carrying plasmids.

**FIG 4:**
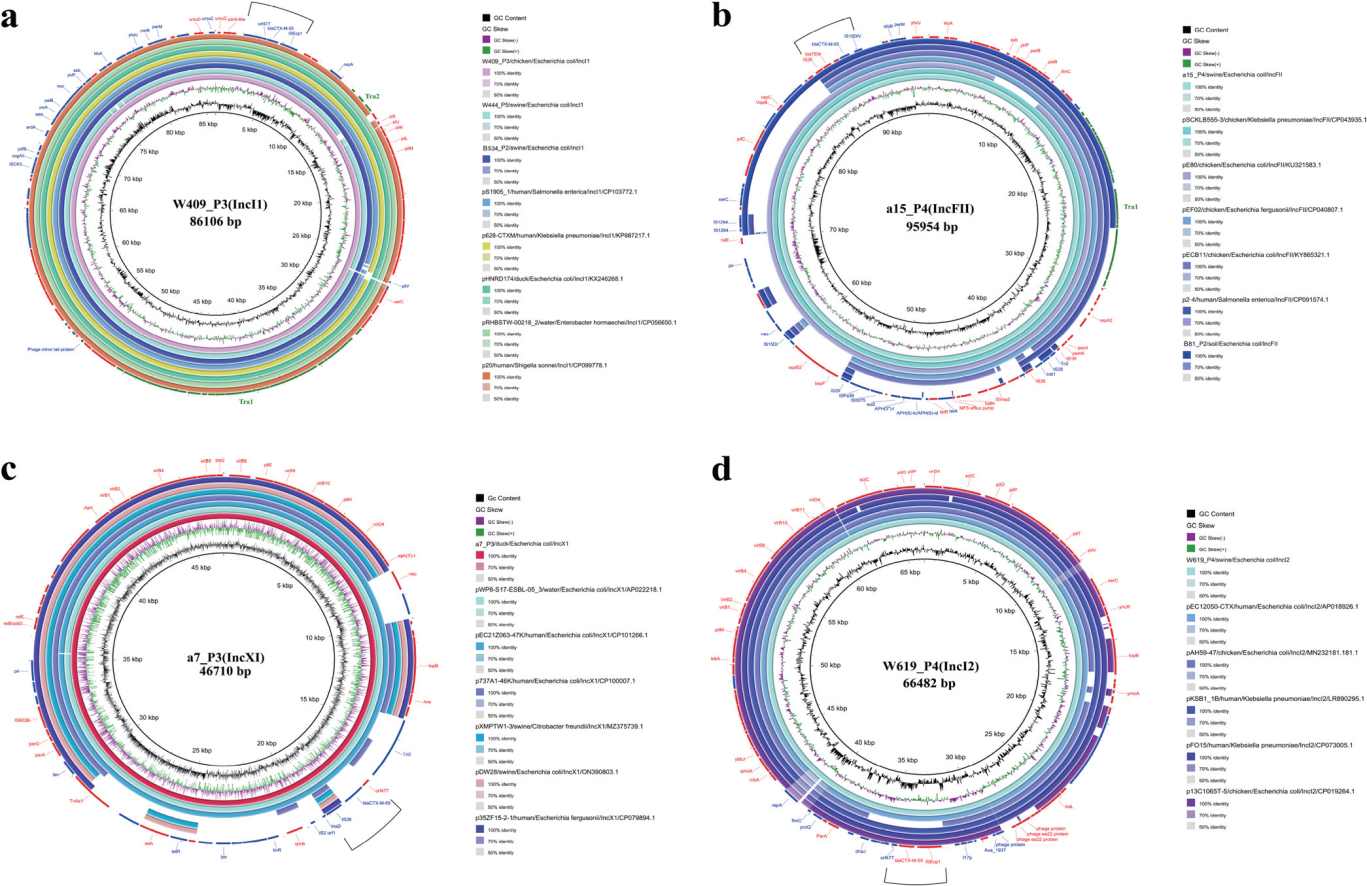
Circular alignment of *bla*_CTX-M-55_-carrying plasmids. GC content and GC skew are indicated in the inner circle. The positions and transcriptional directions of CDs are denoted with arrows. The tra region is colored turquoise. (a) Gene positions and transcriptional directions in the outer circle were derived from W409_P3, which was used as a reference. (b) Gene positions and transcriptional directions in the outer circle were derived from a5_P4, which was used as a reference. (c) Gene positions and transcriptional directions in the outer circle were derived from a7_P3, which was used as a reference. (d) Gene positions and transcriptional directions in the outer circle were derived from W619_P4, which was used as a reference.

Based on the above results and the information of the laboratory preserved strains, we subjected 7 isolates to long-read Nanopore sequencing with plasmid types IncI1 (*n* = 3), IncI2 (*n* = 1), IncX1 (*n* = 1) and IncFII (*n* = 2). Among them, the three IncI1-type plasmids (W409_P3, W444_P5, and B534_P2) were highly similar (>95%), with a size of ~86 kb, and they exhibited distinct ST differences, with ST746, ST3288 and ST485, respectively. Mapping each of the four different plasmid types to the NCBI database (≥98% similarity) showed that both IncI1 and IncI2 have a high degree of similarity to other reference sequences in the NCBI database (from different hosts, including Salmonella enterica, Klebsiella pneumoniae, and Enterobacter hormaechei; from different sources, including human, duck, chicken, swine, and water) and that they both contain the same gene structure “IS*Ecp1*-*bla*_CTX-M-55_-*orf477*” (Fig. 4a and d). Therefore, we hypothesize that IncI1 and IncI2 are important for the wide spread of *bla*_CTX-M-55_ due to their wide host range and ability to persist stably in different sources. Equally important, the plasmid-binding transfer coding region (Tra) was found in both W409_P3 (IncI1) and a15_P4 (IncFII). Although the plasmid structures of a15_P4 and B81_P2 are not similar, the same gene structure, “IS*26*-*bla*_TEM_-*hp*-*bla*_CTX-M-55_-IS*26*(IS*15DIV*),” is still present. In addition, *bla*_CTX-M-55_ is present in the plasmid variable region at a7_P3 (IncX1), where “*orf477*-*bla*_CTX-M-55_” may integrate downstream of IS*26* via Tn2 (Fig. 4b and c).

### Genetic environment surrounding *bla*_CTX−M−55_.

To better explore the origin of *bl*a_CTX-M-55_, the nucleotide sequences of 243 *bla*_CTX-M_ variants and their respective earliest isolation times were collected as completely as possible and inferred by Bayesian evolutionary analysis of sampling trees (BEAST) for temporal divergence estimation. Based on temporal calibration, the clip substitution rate of *bla*_CTX-M_ was inferred to be 0.026/site/year. In addition, *bla*_CTX-M_ may have existed as early as 200 B.C. but rapidly diverged into multiple variants, including *bla*_CTX-M-55_, around the end of the 20th century (Fig. S9 and Table S5).

**FIG 5:**
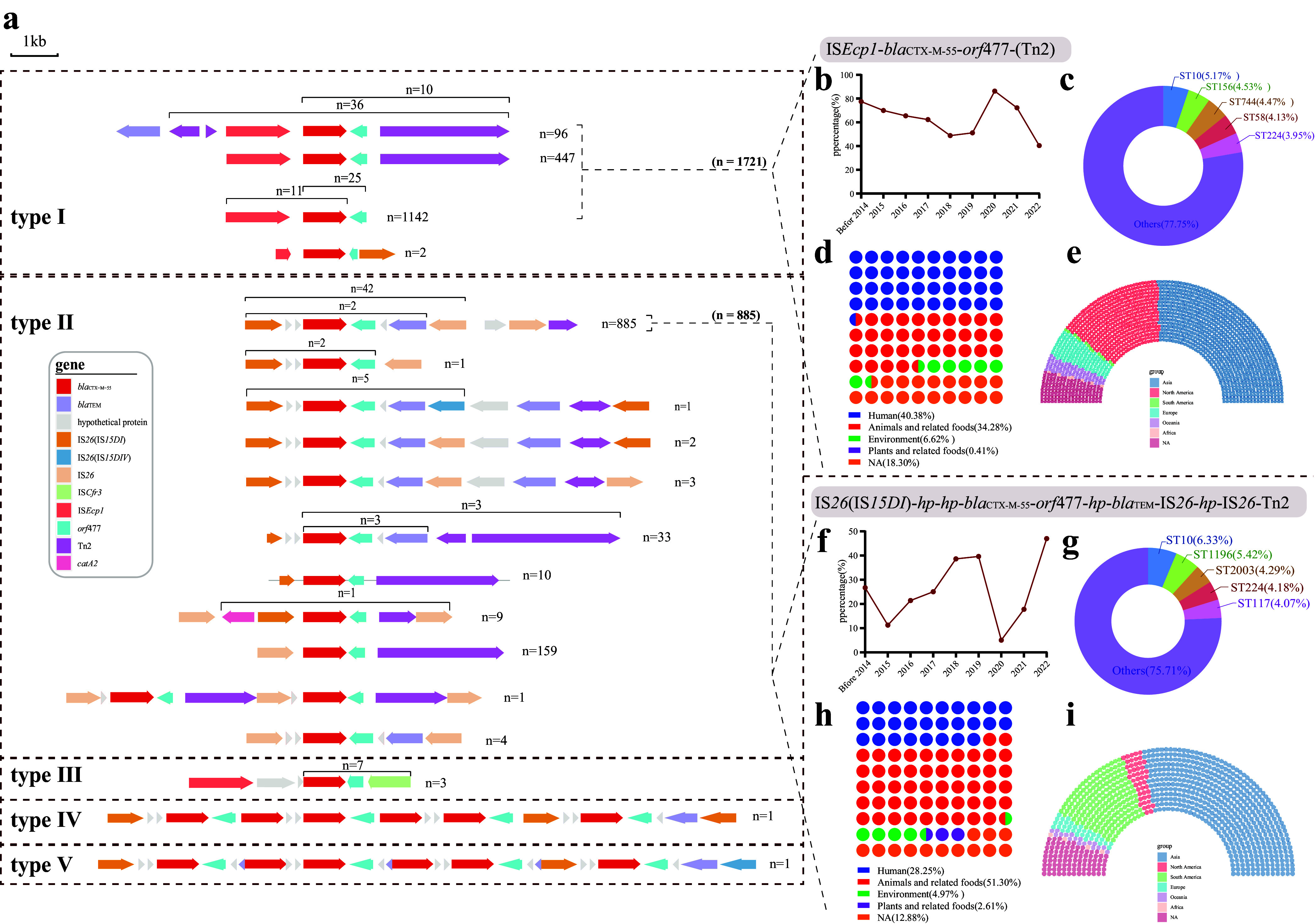
Genetic environment of *bla*_CTX-M-55_-flanking genes and their distribution characteristics. (a) Genetic environment of *bla*_CTX-M-55_ in 2144 strains of Escherichia coli. Arrows indicate the direction of transcription, and different genes are shown in different colors. (b–e) The trend of “IS*Ecp1*-*bla*_CTX-M-55_-*orf*477-(Tn2)” in the folding line of the percentage in different years and showing the distribution of the percentage for different STs, sample origins, and regions. (f–i) The trend of “IS*26*(IS*15DI*)-*hp*-*hp*-*bla*_CTX-M-55_-*orf477*-*hp*-*bla*_TEM_-IS*26*-*hp*-IS*26*-Tn2” in the folding line of the percentage in different years and showing the distribution of the percentage for different STs, sample origins, and regions.

We identified 2947 *bla*_CTX-M-55_ and flanking environments (<10 kb) among 2144 isolates, and after clustering and deredundancy steps, we finally obtained 5 types of genetic environment structures (I ~ V), the most dominant of which were type I in “IS*Ecp1*-*bla*_CTX-M-55_-*orf477*-(Tn2)” (*n* = 1721, 58.40%) and “IS*26*(IS*15DI*)-*hp*-*hp*-*bla*_CTX-M-55_-*orf477*-*hp*-*bla*_TEM_-IS*26*-*hp*-IS*26*-Tn2” (*n* = 885, 30.03%) in type II (Fig. 5a). The former showed an overall decreasing trend in its proportion over time, and ST10 (5.17%) was the most predominant among them to obtain the ST. In addition, this genetic environment is often found in humans (40.38%), with a higher proportion in Asia (51.19%) and North America (27.48%) (Fig. 5b, c, d, e). The latter shows an overall increasing trend over time, and ST10 (6.33%) is again the most predominant ST and is often found in animals and related foods (51.30%), with a high prevalence in Asia (56.72%) and South America (21.36%) (Fig. 5f, g, h, i). At one end of the genetic structure, type III is a fragment carried by IS*Cfr3*, which accounts for a relatively small percentage (*n* = 10). Notably, types IV and V both have 6 similar copies of *bla*_CTX-M-55_ gene structures, both are carried by IS*26*(IS*15DI*), and both were isolated from humans in Asia (Fig. 5a).

## DISCUSSION

ESBLs have seriously challenged the use of β-lactams in human medicine and animal husbandry (16), of which CTX-Ms is considered the most prevalent ESBL in clinical E. coli worldwide (17). In recent years, *bla*_CTX-M-55_-positive E. coli have been widely reported in clinical patients and farm animals, showing a rapid increase (7, 18), which poses a great risk to human health and agricultural production. Therefore, in this study, the evolution and transmission characteristics of *bla*_CTX-M-55_-positive E. coli were further elucidated by collecting 2144 *bla*_CTX-M-55_-positive E. coli genomes globally through a comprehensive genomic analysis approach to outline some key observations in the epidemiology of *bla*_CTX-M-55_-positive E. coli in a “One Health” context.

Previous studies reported that the high clonotype ST131 was often detected in ESBL-producing E. coli (19), but the results of this study showed that the percentage of ST131 was lower and conversely the percentage of ST10 was the highest and showed an increasing trend across years, which may be caused by the preference of some specific *bla*_CTX-M_ variants for an ST, such as *bla*_CTX-M-27_ with a preference for ST131 (20). Potential sources of ESBL-producing *Enterobacteriaceae* in humans include food, companion animals, and the environment, among other factors (21). Furthermore, it has been shown that *bla*_CTX-M-15_-positive E. coli invade almost all human and animal spaces and undergo extensive exchange diffusion (22). Similarly, the frequent clonal transmission of *bla*_CTX-M-55_-positive E. coli between humans, animals (duck, chicken, and swine) and the environment (water and soil) detected in our study, based on the concept of “One Health,” indicates that drug-resistant isolates will eventually pose a serious potential risk to human public health. It is commonly believed that ESBL-producing E. coli tend to be transmitted from animals to humans through the food chain (23). However, wild birds can be infected with human-derived *bla*_CTX-M-55_-positive E. coli (24). The clonal transmission of pathogenic bacteria from humans in 2014 to chickens in 2016 detected in this study suggests that pathogenic bacteria may be transmitted to animals through human activities or waste.

A Korean study found that a strain of *bla*_CTX-M-55_-positive E. coli isolated from rectal swabs of patients carried both *bla*_NDM-9_ and *mcr-1* (25). In addition, a hybrid plasmid from an IS*26*-mediated E. coli was found to carry both *bla*_CTX-M-55_ and *tet*(X4) (26). This is consistent with the results in the present study, and we found that this phenomenon presents a regular distribution in some specific STs, such as ST167, ST156 and ST3997. Tigecycline is the last line of defense against Gram-negative infections, and unfortunately, the successive emergence of *tet*(X3) and *tet*(X4) mediated by plasmids has limited the clinical effectiveness of such drugs (26). Therefore, the cotransmission of *bla*_CTX-M-55_, *fosA3* and *tet*(X4) in E. coli ST3997 detected in this study may promote the generation of “superbugs,” which need to be monitored in the long term.

Conjugative plasmids are one of the most important mechanisms for the emergence and spread of *bla*_CTX-M_, contributing to the horizontal transfer of *bla*_CTX-M_ to other isolates and even breaking the species barrier (27). The spread of spliceable epidemic plasmids is thought to be the most important factor contributing to the spread of *bla*_CTX-M-55_, mostly reported to be localized to IncFII, IncI1, IncI2, and IncHI2 plasmids (28–30). Our study revealed that IncI1 and IncI2 carrying *bla*_CTX-M-55_ have relatively stable genetic structures in different hosts and different sources. IncI1 and IncI2 may play an important role in the widespread dissemination of *bla*_CTX-M-55_ in *Enterobacteriaceae*. In addition to the epidemic plasmid, horizontal transmission of ESBL genes in animal E. coli is also associated with multiple ISs, such as IS*Ecp1*, IS*CR1*, IS*26,* and IS*10*, transposons such as Tn2 and integrons (31, 32). Among them, IS*Ecp1* is frequently found upstream of *bla*_CTX-M_, and IS*Ecp1* can transpose *bla*_CTX-M_ and act as a strong activator for its high expression (12). We identified the two most prevalent structures from the *bla*_CTX-M-55_ flanking environment, carried by IS*Ecp1* and IS*26* (“IS*Ecp1*-*bla*_CTX-M-55_-*orf477*-(Tn2)” and “IS*26*(IS*15DI*)-*hp*-*hp*-*bla*_CTX-M-55_-*orf477*-*hp*-*bla*_TEM_-IS*26*-*hp*-IS*26*-Tn2,” respectively). Of interest, we found that *bla*_CTX-M-55_ carried by IS*Ecp1* is frequently seen in humans, and the proportion decreases yearly, while IS*26*-carried *bla*_CTX-M-55_ is more easily detected in animals and related foods, and the proportion increases yearly. In recent years, *bla*_CTX-M-55_ carried by IS*Ecp1* has often been truncated by IS*26* (33), which is presumed to be the reason for the above phenomenon. The different flanking sequences may be adapted to different media and enable the large-scale propagation and diffusion of *bla*_CTX-M-55_.

Although the *bla*_CTX-M-55_-positive E. coli genomes was collected for the first time in this study on as large a scale as possible, this work still has noteworthy limitations and we must take into account the preferential nature of genomic data sequencing uploads, which may have an impact on our results.

**Conclusion.** In summary, we constructed a global genomic data set of *bla*_CTX-M-55_-positive E. coli for the first time and explored the evolutionary and transmission characteristics of *bla*_CTX-M-55_-positive E. coli by bioinformatics means. Our results reveal that *bla*_CTX-M-55_-positive E. coli is widely distributed globally, especially in Asia. The high ST diversity and the high percentage of auxiliary genomes indicate its high openness. In addition, InclI1, InclI2, IS*Ecp1*, and IS*26* may play important roles in the wide spread of *bla*_CTX-M-55_ in different sources and different hosts. Overall, these results suggest a potential risk of the rapid spread of *bla*_CTX-M-55_-positive E. coli, which reminds us of the need to enhance long-term continuous surveillance of *bla*_CTX-M-55_-positive E. coli.

## MATERIALS AND METHODS

### Sample collection and whole-genome sequencing.

We collected a total of 66 strains of *bla*_CTX-M-55_-positive E. coli from different sample sources (duck, water, snail, soil, pig, and chicken) in China from 2002 to 2021, from our laboratory conserved strains. DNA was extracted from 66 E. coli strains in this study using the HiPure Bacterial DNA kit (Magen) according to the manufacturer's instructions. Sequencing libraries were constructed using the NEXT Ultra DNA Library Prep kit (New England Biolabs, Beverley, MA, USA) and then sequenced using the Illumina HiSeq 2500 platform. The clean reads were assembled using CLC Genomics Workbench (Version 10.0.1, CLC Bio, Aarhus, Denmark), retaining contigs of more than 500 bp. To understand the characteristic distribution of the plasmids carrying *bla*_CTX-M-55_, seven isolates were selected for long-read sequencing on a PacBio RSII instrument.

In addition, to more comprehensively demonstrate the transmission pattern and evolution of *bla*_CTX-M-55_-positive E. coli, we collected E. coli genomes from the NCBI GenBank database (as of September 2022, including Contig, Scaffold, and Complete levels) whenever possible and screened them for BLASTP comparison with *bla*_CTX-M55_ amino acid sequences, retaining genomes with a threshold of 100% similarity and 100% coverage. Finally, 2,078 *bla*_CTX-M-55_-positive E. coli genomes were screened. Overall, during the downstream analysis, we collected 2,144 *bla*_CTX-M-55_-positive E. coli genomes. Detailed background information is presented in Table S1.

### Genome annotation.

The sequences of ARGs, plasmids and VFs in the isolates were identified by using ABRicate (v1.0.1) based on the Comprehensive Antibiotic Resistance Database (CARD) (34), and virulence factor database (VFDB, http://www.mgc.ac.cn/VFs/), respectively (e value ≤ 1 × 10^−5^, similarity ≥ 80% and query coverage ≥ 80%) (35). Similarly, ISs were identified by BLASTN according to the ISfinder database (36). multilocus sequence typing (MLST) was performed by mlst (v2.11) (https://github.com/tseemann/mlst) (37), and the minimal spanning tree of the MLST was generated by Phyloviz (38) (http://www.phyloviz.net/). The localization (chromosome or plasmid) of *bla*_CTX-M-55_ in each genome was predicted using RFPlasmid (v0.0.18) with an *a posteriori* probability cutoff value of 0.5 (39).

To better understand the characteristics and evolutionary patterns of *bla*_CTX-M-55_ flanking sequences, we extracted 10 kb of sequences upstream and downstream of *bla*_CTX-M-55_ (40) and then performed cluster analysis using cd-hit-est (v4.7) (identity > 95%, overlap > 90%) (41). After that, manual collation was used to further categorize them, and the obtained sequences were annotated with coding sequences (CDs) by Prokka (v1.14.6) and RAST (v2.0) (42, 43) and verified by comparing with the NCBI nonredundant protein sequence database (NR) using BLASP.

### Phylogenetic analysis.

Core genomic single nucleotide polymorphisms (SNPs) of 878 Chinese isolates were constructed by using snippy (v4.6.0) (https://github.com/tseemann/snippy), followed by prediction and further filtering of recombination imports using Gubbins (v2.4.1) (44). Maximum likelihood trees were constructed by RAxML (GTRGAMMA alternative model) for filtered core-based SNPs (45). Pairwise differential genetic diversity based on SNP number was also calculated by snp-dists (v0.8.2) (https://github.com/tseemann/snp-dists).

### Construction of the pangenome and molecular clocks.

A total of assembled sequences were predicted and annotated with CDs using Prokka (v1.14.6), followed by a pangenomic pipeline analysis using Roary (v3.11.2) (46). The openness and conservativeness of the pangenome was determined by “Heaps' Law” in the R package “Micropan” (https://github.com/larssnip/micropan.git). If alpha < 1.0 the pangenome is open; if alpha > 1.0 it is closed. Bayesian temporal estimates were inferred from phylogenetic analysis of 243 *bla*_CTX-M_ variants by BEAST (v1.10.4) (47). The models are selected by filtering through the “MODELS” module in the MEGA11, and the best Bayesian Information Criterion (BIC) corresponding module is selected for subsequent analysis. Evolutionary rates and tree topology were analyzed using the TN93 model and general-time reversible (GTR) model, which has 4 rate classes for intersite rate variation. Since the evolutionary rate during the evolution of 243 *bla*_CTX-M_ variants is not always constant, we chose the uncorrelated relaxed clock statistical model for subsequent analysis based on the principle and chose the constant size prior as the tree prior. The earliest separation time for each sample was collected for calibration, and Bayesian Markov chain Monte Carlo (MCMC) was run for 400 million generations with sampling every 40,000 generations. Convergence was assessed using Tracer (v1.7.2) (48), with the first 10% of each chain discarded as aged and ensuring that all relevant parameters reached an effective sample size (ESS) of over 200. The final tree files were exported by TreeAnnotator (v1.10.4) (49).

### Statistical analysis and graphing.

Principal coordinate analysis (PCoA) was performed based on Bray–Curtis distances using the “vegan” package in R software (v4.1.3). In addition, the “vegan” package was used to analyze the alpha diversity index (Shannon). The Wilcoxon test was used to assess the differences between the data sets by using the “ggpubr” package, the results of which are presented in the raincloud plots. *P* < 0.05 was considered to indicate statistical significance. ARG abundance was log_2_ transformed and visualized in a heatmap (“pheatmap” package). Gene structure comparisons were performed by the “gggenes” package, and linear correlations were visualized by the “ggstatsplot” package. All phylogenetic trees were annotated with the online tool Interactive Tree of Life (iTOL v6.5.7) and edited for aesthetic purposes. The network was explored and visualized using Gephi (v0.9.2). Plasmid comparison circle maps were constructed by BRIG (v0.95). Graphs such as line graphs and pie charts are drawn by the “ggplot2” package in GraphPad Prism 8. Simple graphs such as maps and stacked graphs were created by Hiplot (https://hiplot.com.cn).

### Data availability.

The whole-genome sequence generated in this study is available from the National Center for Biotechnology Information (Accession No. PRJNA954068).
