# Supplementary material for: Genomic Insights into Global blaCTX-M-55-Positive Escherichia coli Epidemiology and Transmission Characteristics
Source: Microbiol Spectr. 2023 Jun 26;11(4):e01089-23. doi: 10.1128/spectrum.01089-23 (PMC10434037; doi:10.1128/spectrum.01089-23)
Supplement: Supplemental file 1 — Fig. S1-S9. Download spectrum.01089-23-s0001.docx, DOCX file, 2.4 MB [file spectrum.01089-23-s0001.docx]

**Appendix**

Supplementary material

**Genomic insights into global *bla*_CTX-M-55_-positive** ***[Escherichia coli](javascript:;)* epidemiology and transmission characteristics**

Jin-Tao Yang^† a, b^, Li-Juan Zhang^† c^, Yang Lu ^a, b^, Rong-Min Zhang^a, b^, Hong-Xia Jiang* ^a ,b^

^a^Guangdong Laboratory for Lingnan Modern Agriculture, Guangzhou, China, South China Agricultural University, Guangzhou, 510642, China;

^b^Guangdong Key Laboratory for Veterinary Pharmaceutics Development and Safety evaluation, College of Veterinary Medicine, South China Agricultural University, Guangzhou, 510642, China;

^c^Zhaoqing Branch Center of Guangdong Laboratory for Lingnan Modern Agricultural Science and Technology, Zhaoqing 526000, China

^†^ These authors contributed equally to this work.

^*^ For correspondence: Hong-Xia Jiang, Ph.D.

Guangdong Provincial Key Laboratory of Veterinary Pharmaceutics Development and Safety Evaluation, College of Veterinary Medicine, South China Agricultural University (SCAU), 483 Wushan Road, Guangzhou, 510642, China.

Tel.: +86-020-85284896; Email address:

hxjiang@scau.edu.cn.


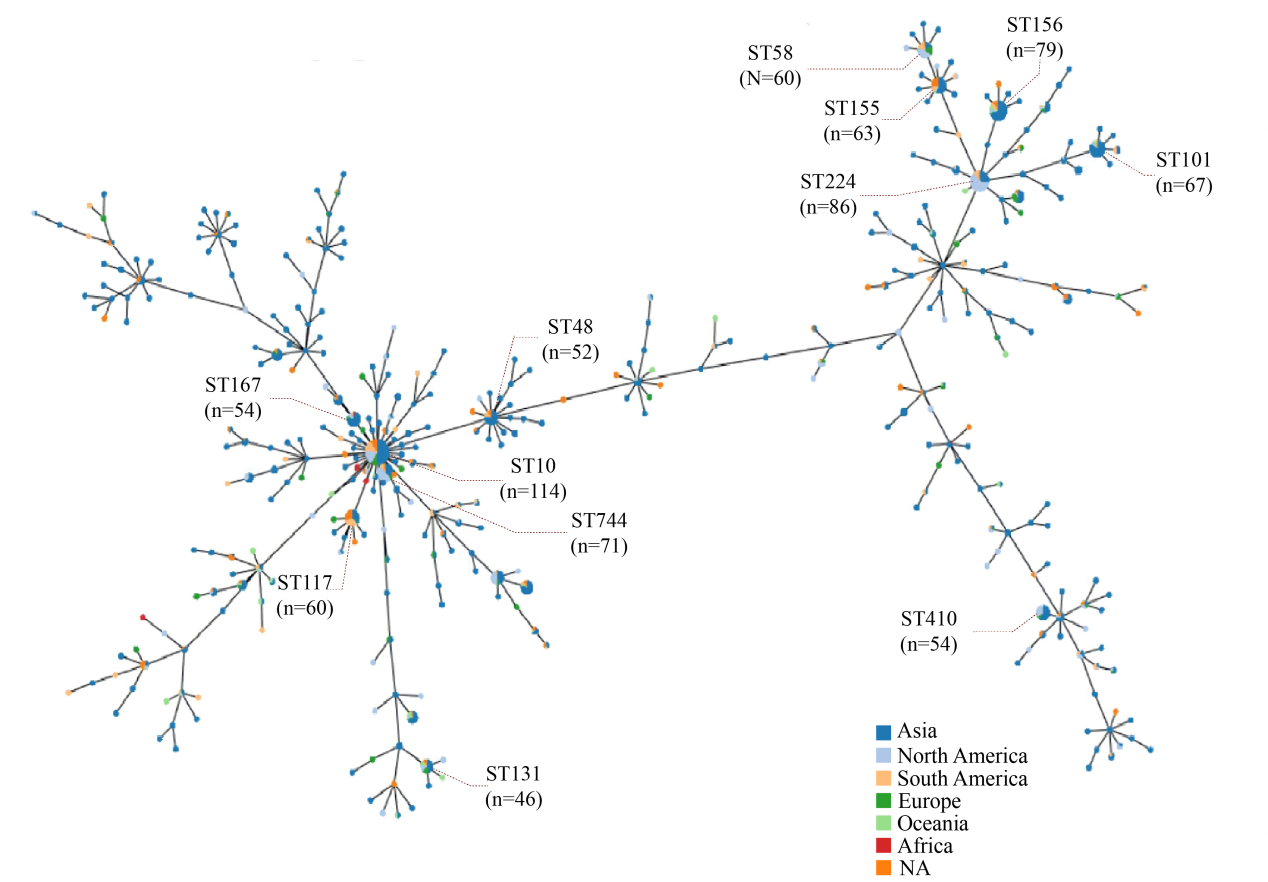


**Figure S1. Minimum spanning tree of the whole dataset by multilocus sequence typing. The region group of all isolates is indicated by different colors.**


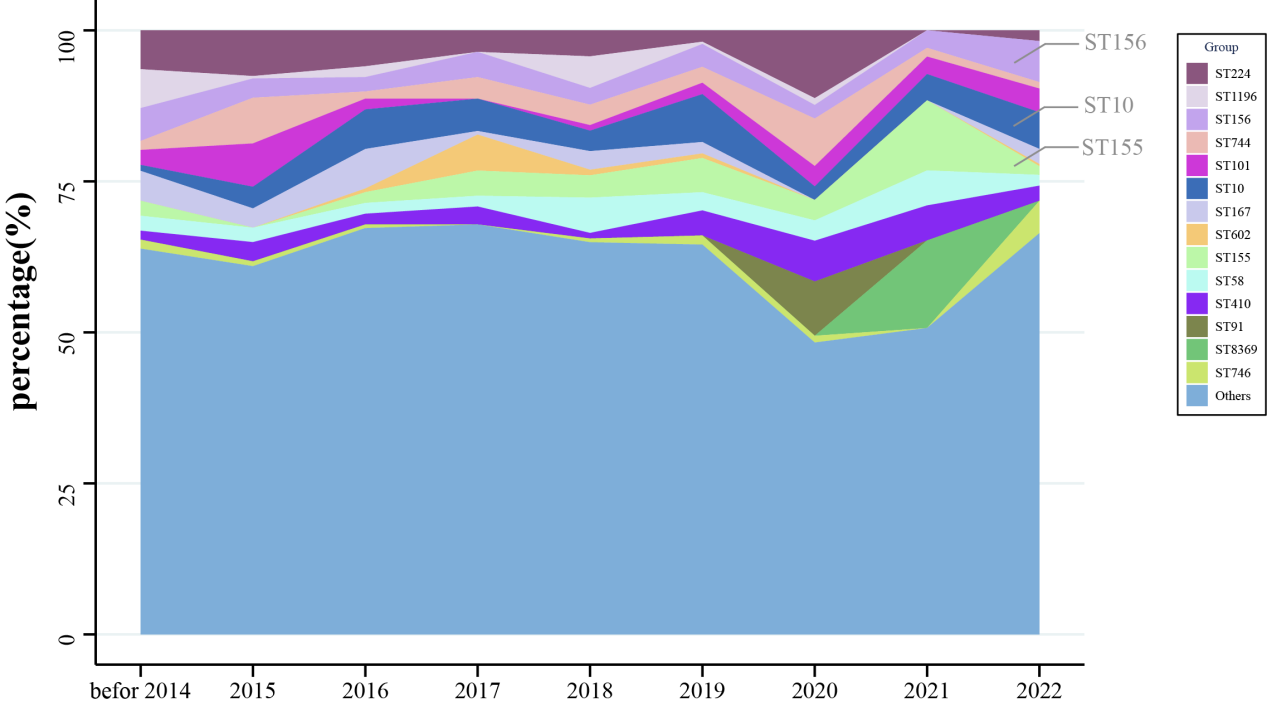


**Figure S2. Trends in STs distribution per year. Since fewer data were collected before 2014, they were combined for analysis.**


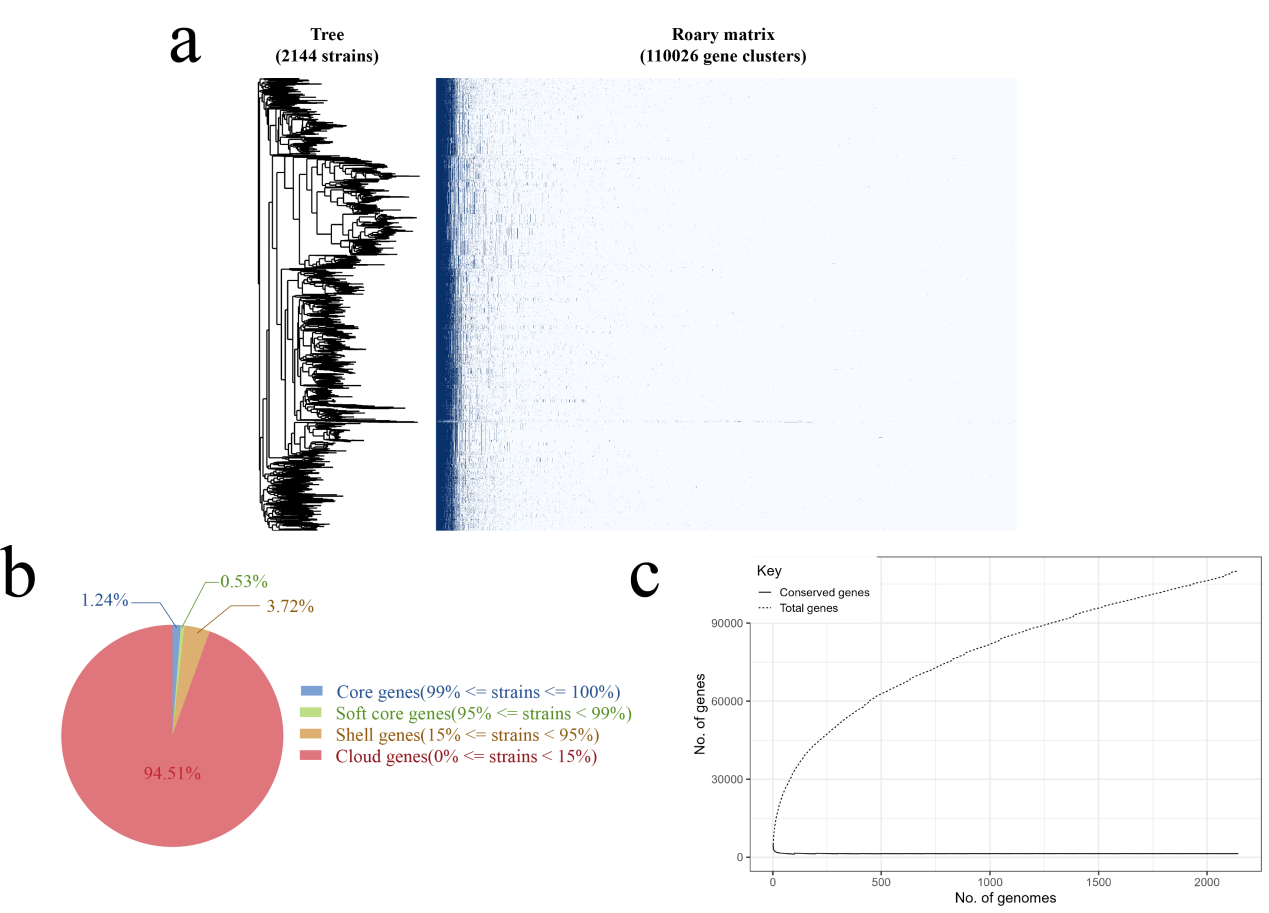


**Figure S3. Pangenomic analysis of 2144** ***bla*_CTX-M-55_-positive *E. coli*** **genomes.** (a) Pangenomic distribution characteristics. (b) Proportions of accessary genes and core genes in the pangenome. (c) Accumulation of the pan genes and core genes with increasing genome number.


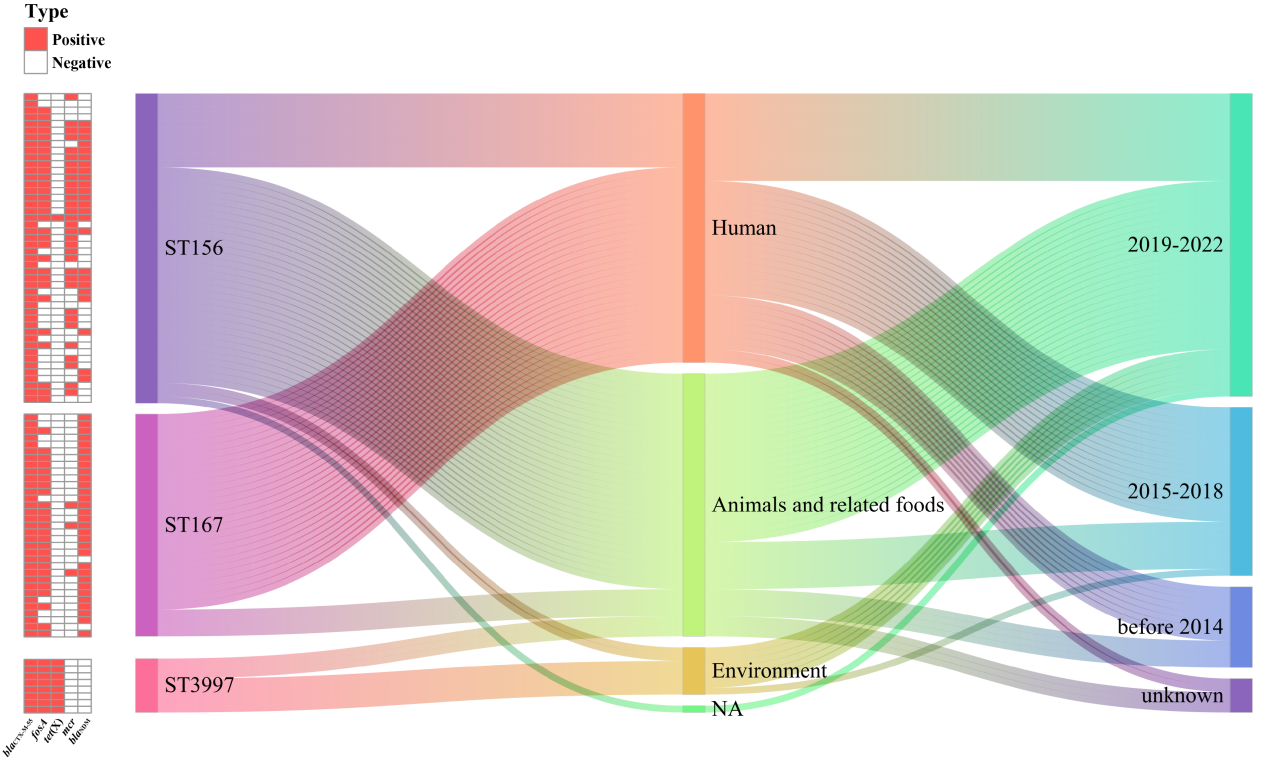


**Figure S4. The characteristic distribution of the three STs (ST156, ST167, ST3997) and their *fosA*, *tet*(X), *mcr*, and *bla*_NDM_ carriage.**


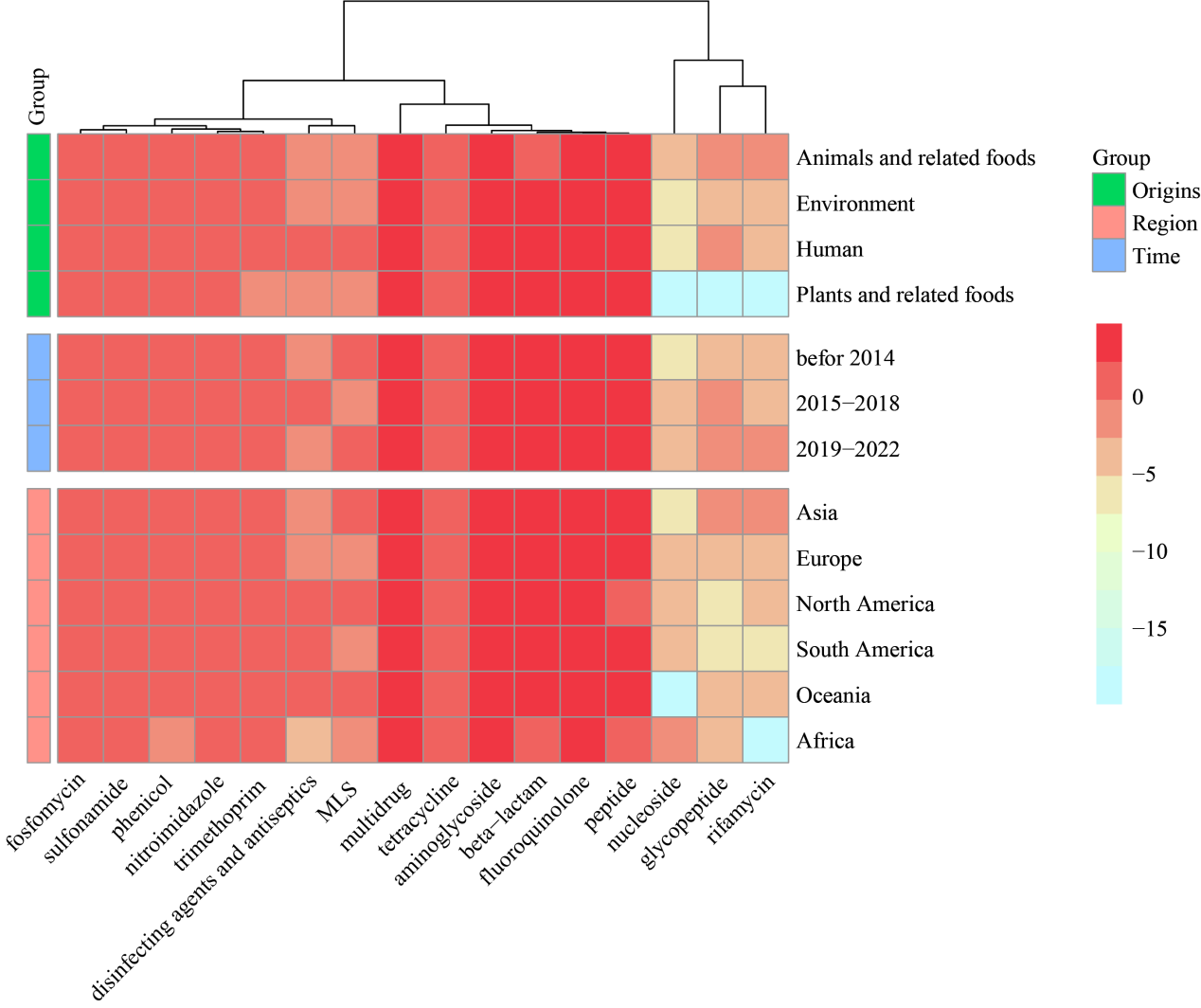


**Figure S5. Broad-spectrum quantitative profile of the ARG types (log2 transformed).**


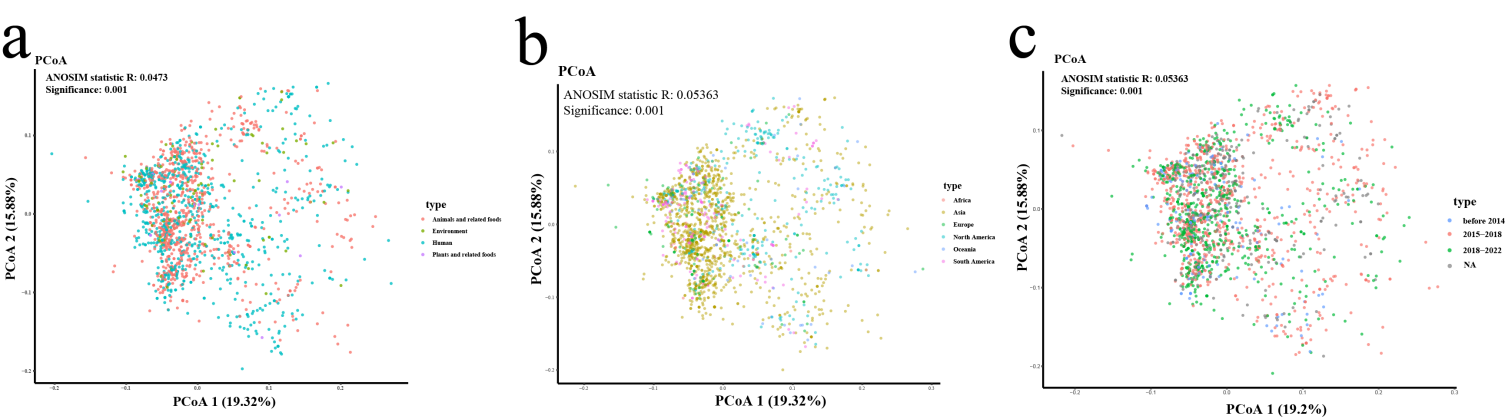


**Figure S6. PCoA and ANOSIM of ARG subtype abundances.**


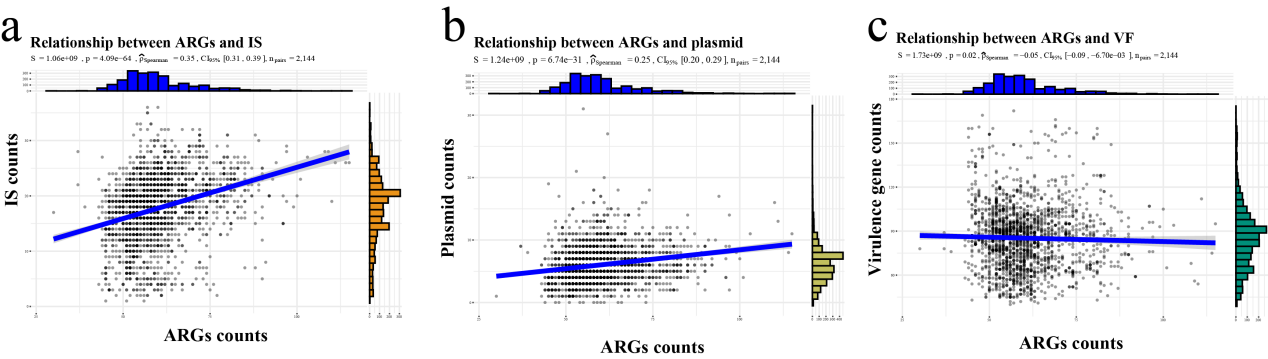


**Figure S7. Potential correlates with the relative abundance of ARGs.** (a) Correlation between the relative abundance of ARGs and the relative abundance of ISs. (b) Correlation between the relative abundance of ARGs and the relative abundance of plasmids. (c) Correlation between the relative abundance of MGEs and the relative abundance of VFs.


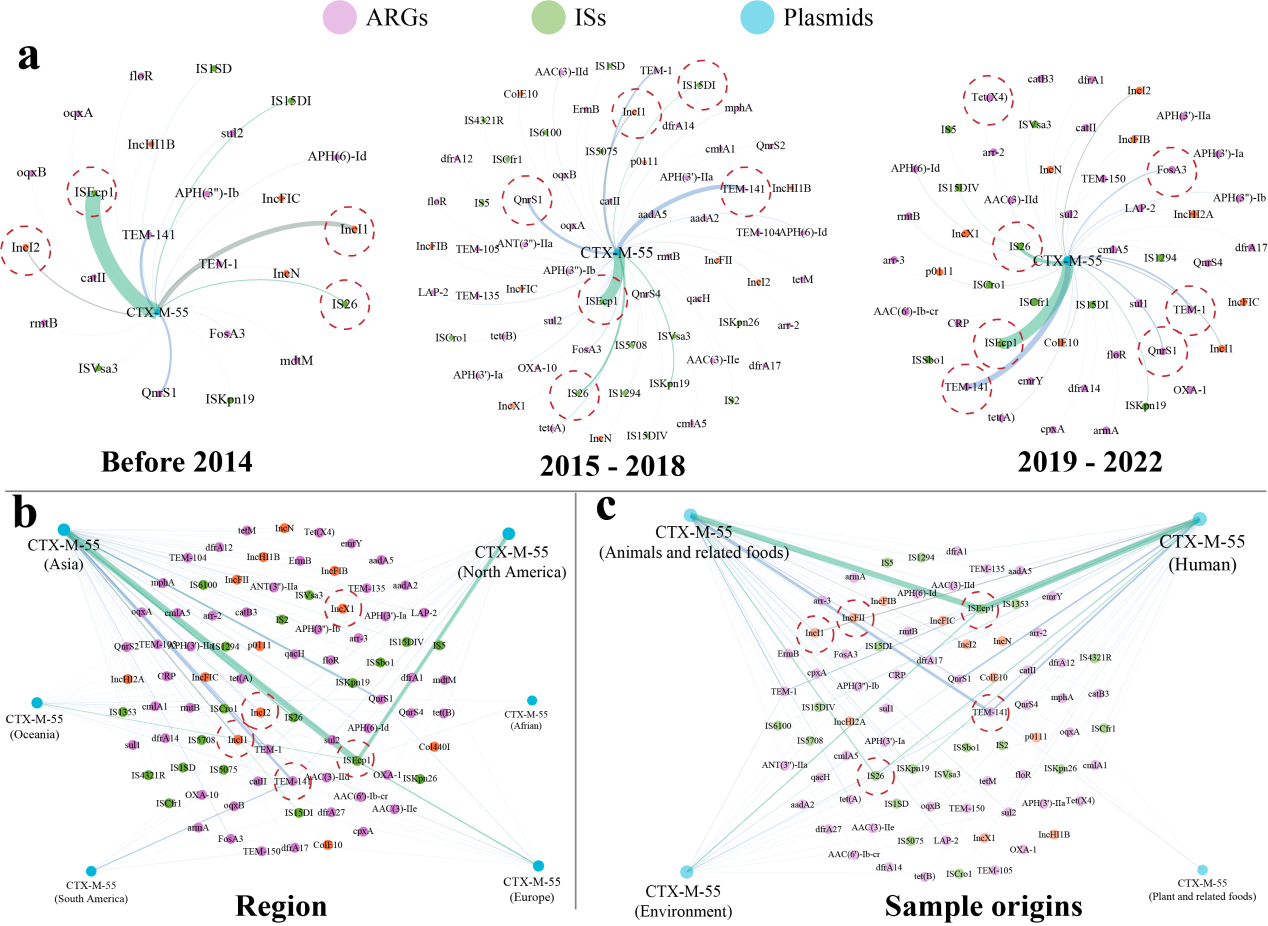


**Figure S8. Cooccurrence network of ARGs, ISs and plasmids associated with *bla*_CTX-M-55_.**The nodes are colored according to classification. Pink represents ARGs, green represents ISs, and blue represents plasmids. (a) Displayed by time group. (b) Displayed by region group. (c) Displayed by sample origin group.
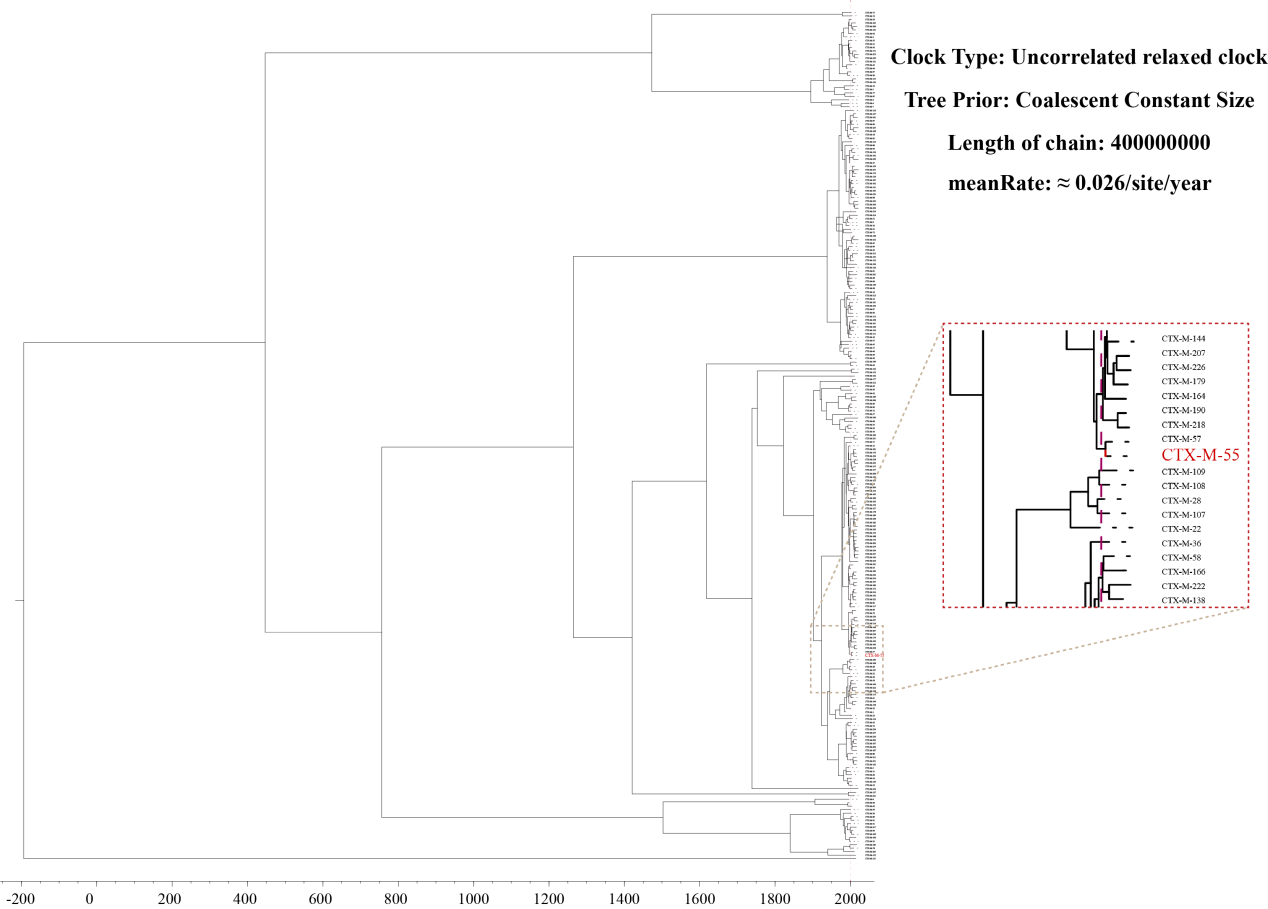


**Figure S9. Bayesian time trees of 243 *bla*_CTX-M_ variants.**
